# Association between *P16^INK4a^* Promoter Methylation and HNSCC: A Meta-Analysis of 21 Published Studies

**DOI:** 10.1371/journal.pone.0122302

**Published:** 2015-04-02

**Authors:** Hao Shi, Xiong Chen, Cheng Lu, Changmei Gu, Hongwei Jiang, RuiWei Meng, Xun Niu, Yangxin Huang, Meixia Lu

**Affiliations:** 1 Department of Epidemiology and Biostatistics, and the Ministry of Education Key Lab of Environment and Health, School of Public Health, Tongji Medical College, Huazhong University of Science and Technology, Wuhan, Hubei, China; 2 Department of Otolaryngology, Union Hospital, Tongji Medical College, Huazhong University of Science and Technology, Wuhan, Hubei, China; 3 Department of Anatomy, Medical College of Nanchang University, Nanchang, Jiangxi, China; 4 Department of Epidemiology and Biostatistics, College of Public Health, University of South Florida, Tampa, FL, United States of America; Karolinska Institutet, SWEDEN

## Abstract

**Background:**

The *p16^INK4a^* is an important tumor suppressor gene (TSG) and aberrant methylation of promoter is known to be a major inactivation mechanism of the tumor suppressor and tumor-related genes. Aberrant TSG methylation was considered an important epigenetic silencing mechanism in the progression of head and neck squamous cell carcinoma (HNSCC). However, some studies have reported differences in the methylation frequencies of *P^16INK4a^* promoter between cancer and the corresponding control group. Therefore, we conducted a meta-analysis to better identify the association.

**Methods:**

PubMed, Ovid, ISI Web of Science, and EMBASE were searched to identify eligible studies to evaluate the association of *p16^INK4a^* promoter methylation and HNSCC. Odds ratio (ORs) and 95% confidence intervals (95%CI) were calculated to evaluate the strength of association between *p16^INK4a^* promoter methylation and HNSCC.

**Results:**

A total of twenty-one studies with 1155 cases and 1017 controls were included in the meta-analysis. The frequencies of *p16^INK4a^* promoter methylation in the cancer group were significantly higher than those in the control group (cancer group: median: 46.67%, range = 7.84%-95.12%; control group: median: 18.37%, range = 0–83.33%; respectively). The pooled odds ratio was 3.37 (95%CI = 2.32–4.90) in the cancer group versus the corresponding control group under the random-effects model.

**Conclusion:**

This meta-analysis of 21 published studies identified that aberrant methylation of *p16^INK4a^* promoter was found to be significantly associated with HNSCC.

## Introduction

Head and neck squamous cell carcinoma (HNSCC) occurs in the oral cavity, oropharynx, hypopharynx and larynx. HNSCC is the sixth most common cancer worldwide and the fifth leading cause of cancer death. Over 500,000 HNSCC cases occur each year [1]. However, the five-year survival of patients with HNSCC was only 40–50% [2].

The pathogenesis of HNSCC is a multistep and multifactorial complex mechanism containing a variety of genetic and epigenetic abnormalities, signal transduction, apoptosis, angiogenesis, and cell cycle regulation [3]. Epigenetic inactivation of the gene resulted from the methylation of CpG islands in promoters is one of the most frequent events in human tumors. The *p16* ^*INK4a*^ gene plays a key role in cell cycle regulation and is located on chromosome 9p21 which consisting of three exons and two introns, spanning approximately 8.5 kb [4, 5]. It is one of the most frequently altered genes observed in various human tumors [6, 7]. P16 prevents the inactivation of retinoblastoma (Rb) protein by inhibiting the cyclin dependent kinases (CDks) and retinoblastoma (Rb) pathway plays an important role in apoptosis and cell cycle regulation [8]. More studies have shown that methylation of *p16* ^*INK4a*^ promoter may play an important role in the development of HNSCC.

Some studies have reported differences in the methylation frequencies of *p16* ^*INK4a*^ promoter between cancer and non-cancerous. However, the results are inconsistent. Therefore, the aims of this meta-analysis are to consolidate the available data and to better identify the association between *p16* ^*INK4a*^ promoter methylation and HNSCC.

## Materials and Methods

### Search Strategy

Relevant studies were identified from the online electronic databases (PubMed, Ovid, ISI Web of Science, and EMBASE) using the search terms: (squamous cell carcinoma or cancer) and (oropharyngeal or oropharynx or head and neck or tonsil) AND (p16 methylation). The search was limited to English language paper. The search results were updated until August 20, 2014.

### Study Selection

**Fig 1:**
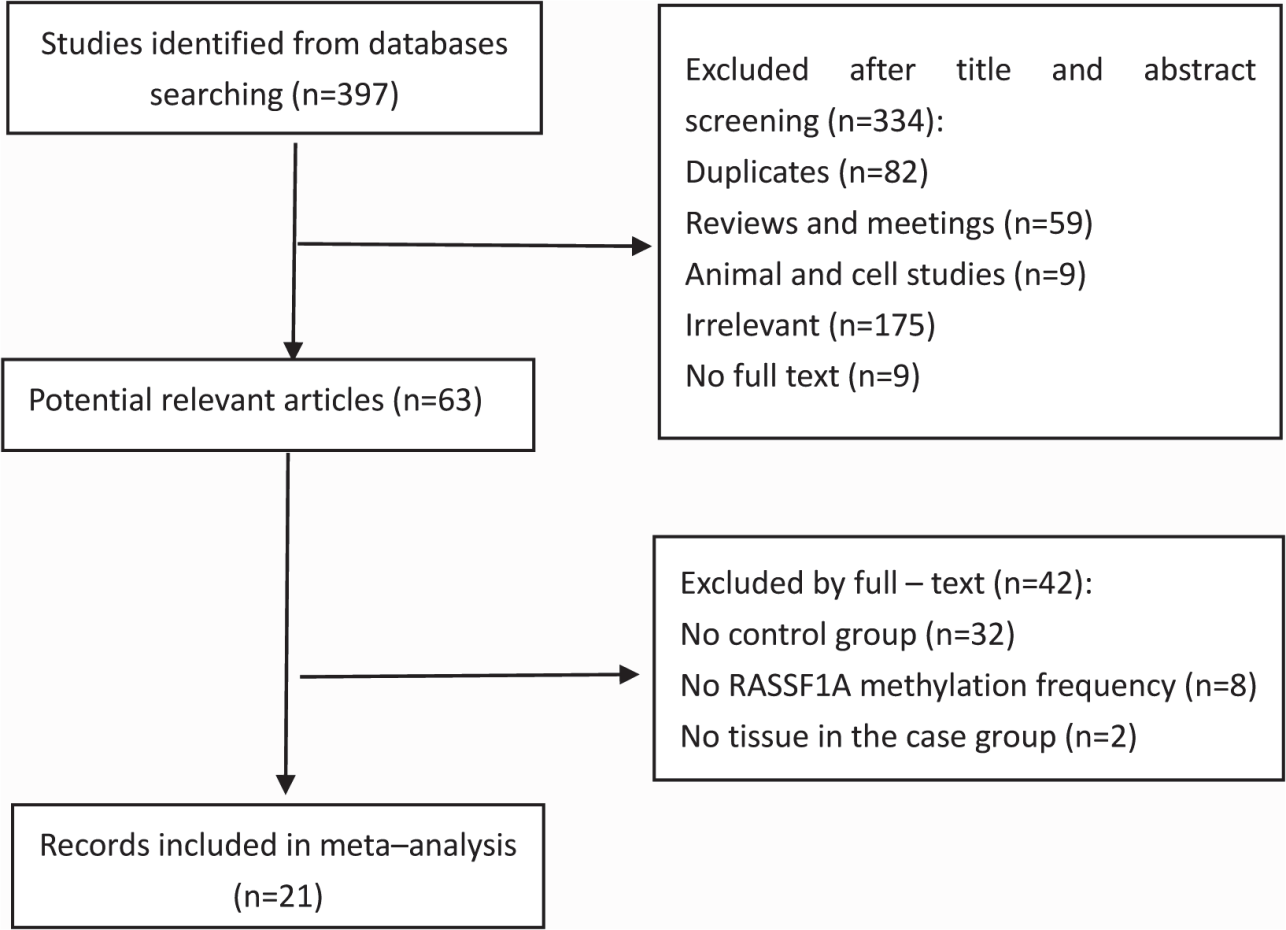
Selection of studies in the meta-analysis.

A study included in the meta-analysis had to meet the following criteria: (1) studies with evaluating the association between *p16* ^*INK4a*^ promoter methylation frequency and HNSCC, (2) case-control study or providing the case and the control cases, (3) providing the *p16* ^*INK4a*^ promoter methylation frequency in case and control groups, (4) specimens of HNSCC were surgically respected primary tumor sample. Firstly, the titles and abstracts of initial searching articles were evaluated for whether it met the inclusion criteria. Then all potentially relevant articles were evaluated on full-text paper. If the results of a study were published more than once, only the most complete and up-to-date information were included in the meta-analysis. The study selection process was shown in Fig 1. Finally, a total of 21 studies (PubMed 13, Web of Science 7, Ovid 1) which contain 1155 cases and 1017 controls were included in our meta-analysis.

### Data Extraction and Quality Assessment

Three reviewers (Hao Shi, Changmei Gu, and Ruiwei Meng) independently reviewed the included articles. The following information was extracted from the included studies: first author’s name, year of publication, study population, sample size, sample type in case and control group, the state of the individual of the control group, the number of people with *p16* ^*INK4a*^ methylation in the case and control groups, the number of the case and control groups. All the detailed information of the included studies was checked by three reviewers (Meixia Lu, Xiong Chen, and Cheng Lu) as described in the Cochrane Handbook for systematic reviews.

### Statistical Methods

The pooled odds ratios (ORs) and their 95% confidence intervals (CIs) were used to assess the strength of the association between *p16* ^*INK4a*^ promoter methylation and HNSCC. The *x* ^2^-based Cochran Q statistic test and *I* ^2^ statistics were used to test the between-study heterogeneity [9]. When *P*<0.05 for the Q statistic or *I* ^2^ ≥50%, the heterogeneity was considered significant and a random-effects model was used to calculate the pooled ORs. Otherwise, a fixed-effects model was applied. The meta-regression (method = restricted maximum-likelihood estimator) was employed to explore the source of heterogeneity. And the subgroup analysis was performed for further evaluation of the source of heterogeneity. The contribution of each study to the final results of the meta-analysis was evaluated according to the sensitivity analysis. Begg’s funnel plot [10] and Egger’s test [11] were used to assess the publication bias. The fail-safe number [12] was also employed to assess the publication bias. In the study, all the *P* values are two sides with a significant level at 0.05. When the individual studies have cells with zero counts, the default is to add 0.5 to all zero counts in the Meta package. All statistical analyses were performed with the Meta package (version 3.0–1; http://CRAN.R-project.org/package=meta) in R (version 3.10; http://www.r-project.org/).

## Results

### Study Characteristics

The comprehensive search and selection procedures of literature were displayed in Fig 1 for evaluating the association between *p16* ^*INK4a*^ promoter methylation frequency and HNSCC. A total of 21 studies with 1155 cases and 1017 controls were included in the meta-analysis. 397 studies were initially identified by searching the electronic databases. 63 potentially relevant studies were retrieved for further evaluation after removing 82 duplicated articles, 59 reviews or meeting reviews, 9 cell lines and animal studies, 175 irrelevant articles, and 9 papers that did not have full text versions. 32 studies without a control group, 8 studies without *p16* ^*INK4a*^ promoter methylation data, and 2 studies without tissue in the case group were excluded from full-text review. Finally, 21 studies were included in our meta-analysis. Ten studies were of Asian subjects and eleven studies were of Caucasian subjects. The control group included HNSCC patients, benign disease patients, and healthy people. And the sample type of control group included tissue, serum, blood, and saliva. The methylation detection method of *p16* ^*INK4a*^ in HNSCC and control included 16 studies used methylation-specific polymerase chain reaction (MSP), three studies used real-time quantitative MSP (QMSP), one study used PCR-based methylation-sensitive restriction analysis (MSRA), and one study used pyrosequencing (Pyro). Six studies [13–18] were the methylated CpG islands of promoter and fifteen studies [19–33] were the methylation of promoter. Study characteristics were summarized in Table 1.

**Table 1:** Characteristics of studies included in the study characteristics of included studies.

			Case		Control			Control	Control
Author	Year	Region	M	U	M	U	Method	style ^#^	sample types
Kis[19]	2014	Hungary	14	36	3	65	MSP	H	saliva
Bhatia[20]	2014	India	62	14	33	36	MSP	H	tissue
					18	52	MSP	H	blood
Dang[21]	2013	China	6	6	5	25	MSP	H	tissue
Demokan[22]	2011	Turkish	41	19	22	55	QMSP	A	tissue
Wong[23]	2011	Taiwan	43	21	20	64	MSP	H	tissue
Weiss[24]	2011	Germany	4	47	2	29	MSP	H	tissue
Laytragoon[13]	2010	Sweden	39	2	15	3	MSP	A	tissue
Kaur[25]	2010	India	44	48	5	43	QMSP	A	tissue
					0	30	QMSP	H	serum
Su[26]	2010	Taiwan	10	20	3	27	QMSP	A	tissue
Steinmann[14]	2009	Germany	32	22	6	17	MSP	A	tissue
Ghosh[27]	2009	India	9	54	7	33	MSRA	H	tissue
Righini[33]	2007	French	20	70	0	30	MSP	A	tissue
					0	30	MSP	H	saliva
					16	60	MSP	A	saliva
Martone[28]	2007	Italy	4	16	3	8	MSP	A	tissue
Shaw[15]	2006	UK	22	58	1	25	Pyro	A	tissue
Maruya[16]	2004	USA	10	22	1	31	MSP	A	tissue
					2	4	MSP	H	tissue
Kulkarni[29]	2004	India	40	20	30	30	MSP	A	tissue
					0	20	MSP	H	saliva
Wong[30]	2003	China	36	37	5	24	MSP	A	tissue
					4	16	MSP	H	serum
Weber[31]	2003	Germany	16	34	12	30	MSP	H	tissue
Nakahara[17]	2001	Japan	16	16	0	32	MSP	A	tissue
Rosas[32]	2001	USA	14	16	11	3	MSP	A	saliva
					1	29	MSP	H	saliva
Sanchez[18]	2000	USA	26	69	8	18	MSP	A	serum

M: *p16* ^*INK4a*^ promoter methylated; U: *p16* ^*INK4a*^ promoter unmethylated

#: A: Autologous (the control from the HNSCC themselves); H: Heterogeneous (the control from other individuals, including blood, serum, saliva or tissue).

### Combining Results of Included Studies

**Fig 2:**
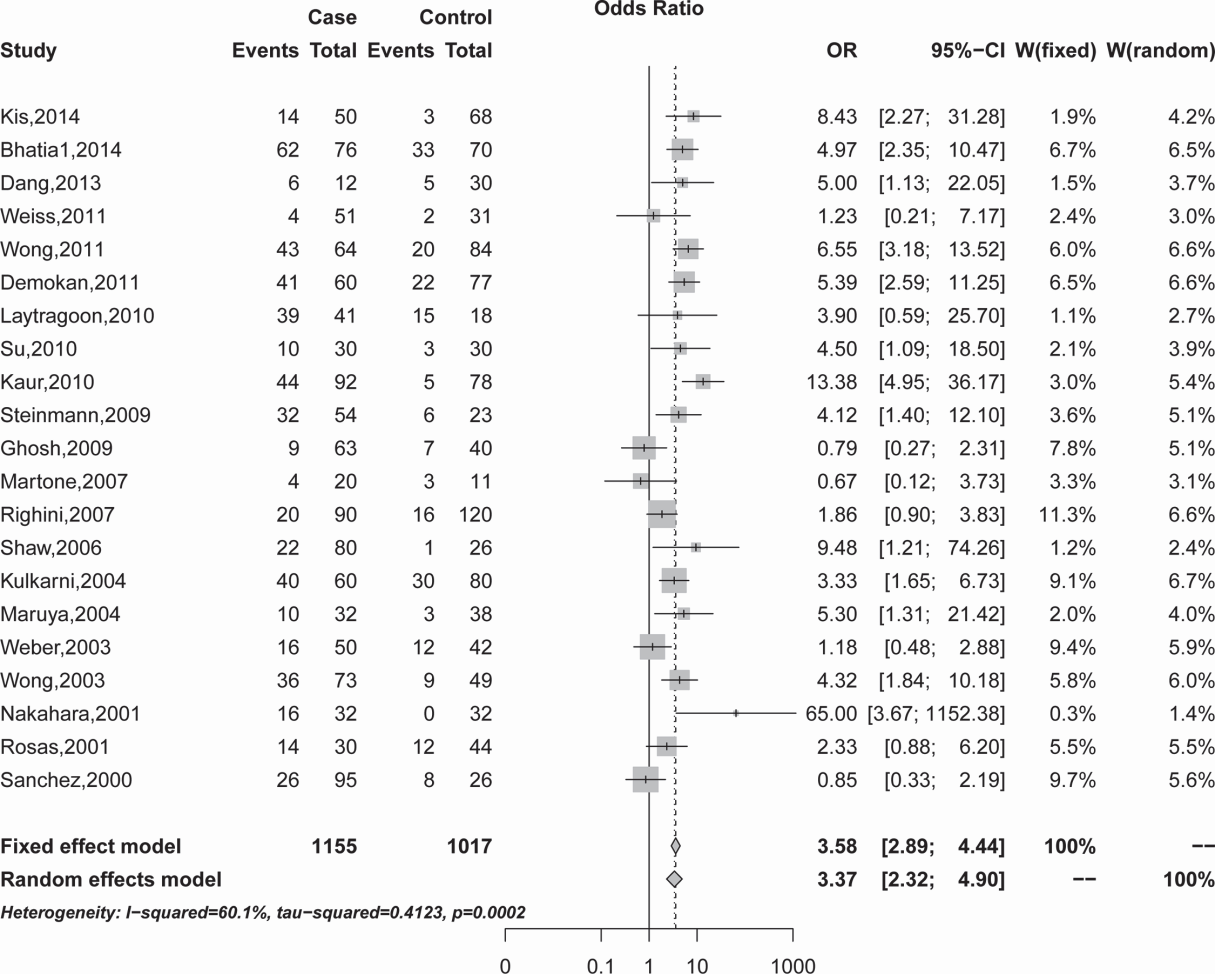
Summary estimates for *p16^INK4a^* promoter methylation frequency associated with HNSCC by meta-analysis.

The combining result showed the association of *p16* ^*INK4a*^ promoter methylation with HNSCC risk in Fig 2. A random-effects model was employed because of the significant heterogeneity was observed among the included studies (*I* ^*2*^ = 60.1%, Q = 50.07, *P*<0.001). In the overall meta-analysis, *p16* ^*INK4a*^ promoter methylation frequency was significantly associated with HNSCC (Summary OR was 3.37, 95%CI = 2.32–4.90) (Fig 2).

### Sensitivity Analysis

A sensitivity analysis was performed by omitting a single study and calculating the pooled OR for the remaining studies under the random-effect model to assess the effects of each individual study on the pooled OR. The results of sensitivity analysis were summarized in the Table 2. According to sensitivity analysis the odds ratio ranged from 3.10 (95%CI = 2.16–4.46) to 3.65 (95%CI = 2.55–5.23) by omitting a single study under the random-effect model. The sensitivity analysis indicated that the pooled OR between *p16* ^*INK4a*^ promoter methylation and HNSCC were reliable and stable.

**Table 2:** Sensitivity analysis of pooled OR for *p16^INK4a^* methylation and HNSCC under the random-effects model.

Study omitted	OR (95%CI)	*P* for heterogeneity	*tau* ^*2*^	*I* ^*2*^ (%)
Kis, 2014	3.27(2.21;4.74)	<0.001	0.41	60.50%
Bhatia, 2014	3.29(2.21;4.89)	<0.001	0.45	61.20%
Dang, 2013	3.32(2.26;4.89)	<0.001	0.43	61.90%
Weiss, 2011	3.48(2.38;5.09)	<0.001	0.42	61.10%
Wong, 2011	3.22(2.18;4.74)	<0.001	0.42	59.20%
Demokan, 2011	3.27(2.20;4.85)	<0.001	0.45	60.70%
Laytragoon, 2010	3.36(2.29;4.93)	<0.001	0.43	62.00%
Su, 2010	3.33(2.26;4.92)	<0.001	0.44	61.90%
Kaur, 2010	3.10(2.16;4.46)	<0.001	0.34	54.90%
Steinmann, 2009	3.34(2.25;4.94)	<0.001	0.45	62.00%
Ghosh, 2009	3.63(2.53;5.22)	<0.001	0.34	55.60%
Martone, 2007	3.54(2.44;5.14)	<0.001	0.39	59.20%
Righini, 2007	3.52(2.38;5.20)	<0.001	0.43	59.70%
Shaw, 2006	3.29(2.25;4.80)	<0.001	0.42	61.20%
Kulkarni, 2004	3.38(2.26;5.06)	<0.001	0.48	62.10%
Maruya, 2004	3.31(2.25;4.87)	<0.001	0.43	61.70%
Weber, 2003	3.59(2.47;5.22)	<0.001	0.37	57.20%
Wong, 2003	3.32(2.23;4.94)	<0.001	0.45	61.80%
Nakahara, 2001	3.23(2.25;4.65)	<0.001	0.37	58.50%
Rosas, 2001	3.45(2.33;5.10)	<0.001	0.44	61.60%
Sanchez, 2000	3.65(2.55;5.23)	<0.001	0.33	54.10%

### Meta-regression and Subgroup Analysis

As the significant heterogeneity across the studies was found (*I* ^*2*^ = 60.1%, Q = 50.07, *P*<0.001), the meta-regression was employed to explore potential sources of heterogeneity. We conducted a multiple regression model with six variables (i.e. Population subgroup, publication year, case sample size, control type, control sample type, and method). No source of significant heterogeneity was found (Table 3). The subgroup analysis was performed to further evaluate the source of the heterogeneity according to populations, case sample size, control type, control sample type, and method.

**Table 3:** Mixed-effects model of Meta-regression analysis.

		95%CI		
Heterogeneity sources	Coefficient	Lower	Upper	*P*
Population	-0.75	-1.74	0.24	0.14
Publication year	0.05	-0.06	0.15	0.39
Case sample size	-0.24	-1.19	0.70	0.61
Method$Pyro	2.09	-0.76	4.93	0.15
Method$QMSP	0.89	-0.43	2.20	0.19
Control style	0.38	-0.58	1.34	0.54
Control sample types	-0.83	-1.81	0.14	0.09

The OR for was 4.76 (95%CI = 3.00–7.54) in Asians under the random-effects model, and 2.25 (95%CI = 1.61–3.13) in Caucasians under the fixed-effects model. With the case sample size, the OR for was 3.33 (95%CI = 1.68–6.60) in the >60 case group under the random-effects model, and 3.59 (95%CI = 2.65–4.87) in the ≤60 case group under the fixed-effects model. In the subgroup analysis of the control type, the frequencies of *p16* ^*INK4a*^ promoter methylation in the autologous group were higher than those in the heterogeneous group including benign disease and healthy individuals under the random-effects model (Autologous: 3.14, 95%CI = 1.76–5.59; Heterogeneous: 4.62, 95%CI = 2.43–8.79; respectively). Subgroup analysis of the control sample type showed that the frequencies of *p16* ^*INK4a*^ promoter methylation in the Non-tissue group (control sample type: serum or saliva) were higher than those in the tissue group (control sample type: tissue) under the random-effects model (Non-tissue: 5.92, 95%CI = 2.94–15.30; Tissue: 3.43, 95%CI = 2.25–5.24; respectively). For the methylation detection method of *p16* ^*INK4a*^ promoter, Ghosh [27] used PCR-based methylation-sensitive restriction analysis (MSRA) and we have put the study classified as MSP group. The OR was 7.30 (95%CI = 4.26–12.48) in the QMSP group under the fixed-effects model, 2.83 (95%CI = 1.89–4.23) in the MSP group under the random-effects model, and 9.48 (95%CI = 1.21–74.26) in the Pyro group. All results of subgroup analysis were summarized in Table 4.

**Table 4:** Subgroup analysis of the association between *p16^INK4a^* promoter methylation and HNSCC.

	Case		Control		M-H pooled OR†	D+L pooled OR‡	Heterogeneity		
Group	M+	N	M+	N	OR (95%CI)	OR (95%CI)	*I* ^*2*^ (%)	*P*	τ^2^
Total	508	1155	215	1017	3.58 (2.89–4.44)	3.37 (2.32–4.90)	60.1	<0.01	0.41
Population subgroup									
Asians	307	562	134	570	4.99 (3.77–6.62)	4.76 (3.00–7.54)	55.3	0.02	0.28
Caucasians	201	593	81	447	2.25 (1.61–3.13)	2.26 (1.39–3.66)	42.8	0.06	0.26
Case sample size									
≤60	246	522	116	524	3.59 (2.65–4.86)	3.38 (2.21–5.17)	37.5	0.08	0.21
>60	262	633	99	493	3.58 (2.65–4.84)	3.33 (1.68–6.60)	77.3	<0.01	0.72
Control style									
Autologous	392	879	126	516	3.12 (2.38–4.08)	3.14 (1.76–5.59)	69.1	<0.01	0.78
Heterogeneous	353	838	90	531	4.93 (3.61–6.72)	4.62 (2.43–8.79)	66.6	<0.01	0.86
Control sample type^$^									
Tissue	463	980	171	687	3.84 (2.99–4.93)	3.43 (2.25–5.24)	55.3	<0.01	0.40
Non-tissue	344	813	63	430	4.54 (3.29–6.25)	5.92 (2.29–15.30)	80.5	<0.01	2.01
Method									
Pyro	22	80	1	26	9.48 (1.21–74.26)	9.48 (1.21–74.26)	-	-	-
QMSP	95	182	30	185	7.30 (4.26–12.48)	7.00 (3.70–13.21)	20.9	0.28	0.07
MSP	391	893	184	806	3.01 (2.38–3.82)	2.83 (1.89–4.23)	58.0	<0.01	0.37

†: the fixed-effects model

‡: the random-effects model

$: non-tissue: serum, saliva and blood

### Publication bias

**Fig 3:**
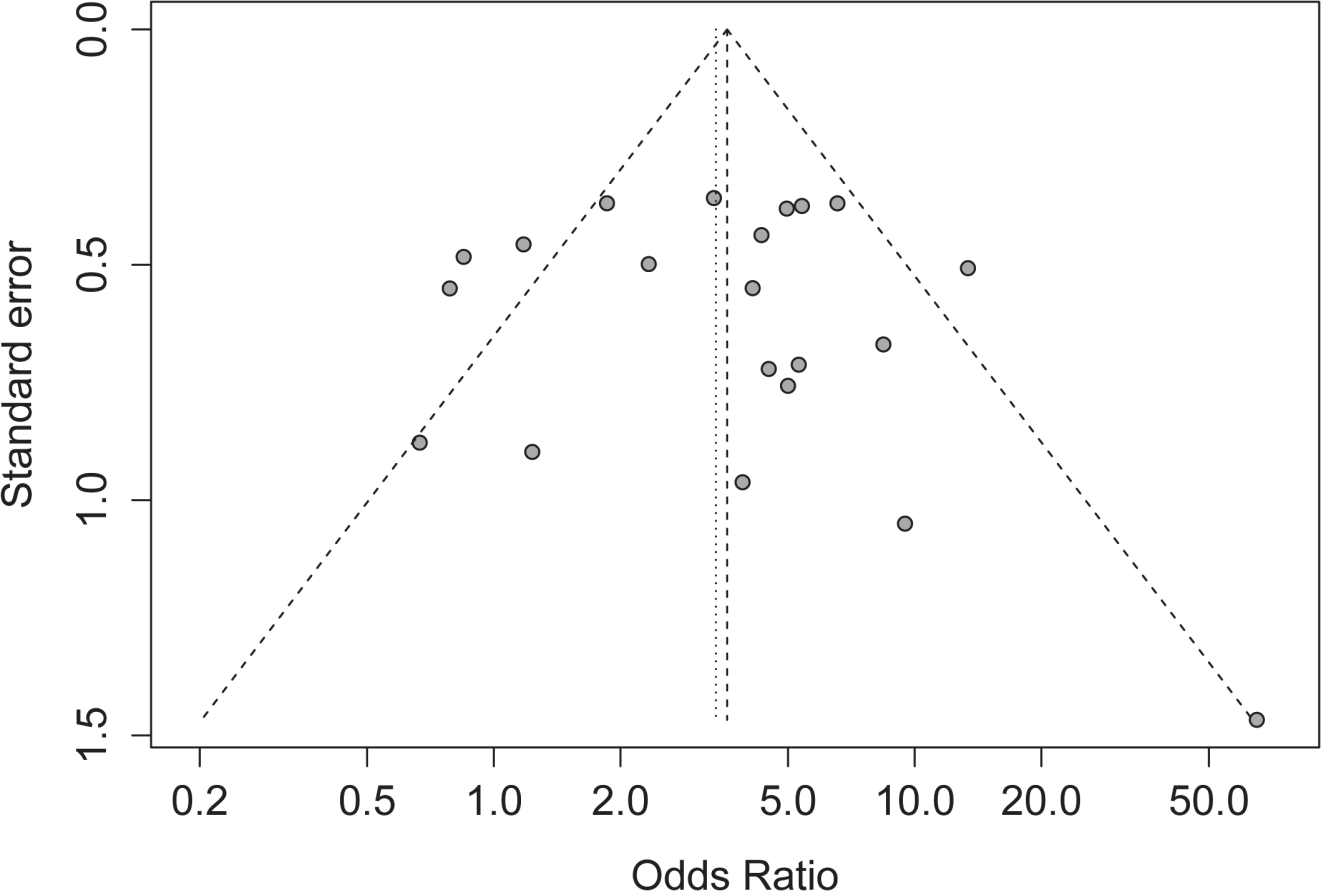
The Begg’s funnel plot for assessment of publication bias in the meta-analysis.

Begg’s funnel plot, Egger’s test, and the fail-safe number were performed to assess the publication bias of the literature. The shape of the Begg’s funnel plot in Fig 3 showed a possible asymmetry, but the Egger’s test did not detect publication bias (*P* = 0.25). The fail-safe number (*Z* = 54.20, N_fs0.05_ = 1071.09, N_fs0.01_ = 520.05) also did not display statistical evidence for publication bias.

## Discussion

Hypermethylation of the tumor suppressor gene (*TSG*) promoter can be found in various cancers and induce the silencing of tumor suppressor genes in the process of carcinogenesis. The *p16* ^*INK4a*^ is an important tumor suppressor and DNA repair genes with a major negative regulator of critical tumor pathway [34] and the pl6/cyclin D/CDK/pRB pathway is also prominent in many epithelial malignancies [35]. Inactivation of *p16* ^*INK4a*^ induced by aberrant hypermethylation plays a role in the process of carcinogenesis in various cancers (i.e. Lung, hepatocellular, gastric, and breast cancer) [36–39].

The current meta-analysis assessed the association between *p16* ^*INK4a*^ promoter methylation and HNSCC, which included 21 studies with 1155 cases and 1017 controls. The frequency of *p16* ^*INK4a*^ promoter methylation in tumor was 43.98% and 21.14% in control. The pooled odds ratio under a random-effect model was 3.37 (95%CI = 2.32–4.90) in the cancer cases versus the controls. The result identified that methylation of *p16* ^*INK4a*^ promoter had 3.37 fold increased risks of HNSCC compared with the control group.

In subgroup analysis, the OR for was 4.76 (95%CI = 3.00–7.54) in Asians under the random-effects model, and 2.25 (95%CI = 1.61–3.13) in Caucasians under the fixed-effects model. The association between *p16* ^*INK4a*^ promoter methylation and HNSCC in Asians was stronger than that in Caucasians. Similarly, Kaur’s study observed that the frequency of *p16* ^*INK4a*^ promoter methylation (47.8%) in Indian cohort was higher than that in North-American cohort (37.5%) [25]. In method subgroup, The OR was 7.30 (95%CI = 4.26–12.48) in the QMSP group under the fixed-effects model, 2.83 (95%CI = 1.89–4.23) in the MSP group under the random-effects model, and 9.48 (95%CI = 1.21–74.26) in the Pyro group. In the past few years, several methods were developed to detect aberrant gene methylation (e.g. MSP, QMSP, Pyro, etc.). MSP is a simple, sensitive, and specific method for detecting the methylation status of CpG rich region [40]. However, MSP and QMSP require particular gene sequence information for the design of PCR primers [41]. The different primers may have an impact on the results. MSP (a nonquantitative nonfluorometric PCR method) could not detect low levels of promoter methylation, unlike QMSP which can detect up to 1/1000 methylated alleles [42]. Pyro is a much more accurate and quantitative method in detecting gene methylation level and is based on DNA sequence analysis. The detection method of gene methylation and primers selected from regions of the same CpG island and gene promoter was different in each study. These factors may have different sensitivities and specificities in detection of gene methylation [43]. In addition, the degrees of heterogeneity of tumors are different, either as infiltrating normal cells or different clones of tumor cells. This will influence the results of methylation analyses. These reasons may lead to different results and be a source of heterogeneity. In the subgroup analysis of sample size, control type, and control sample type, significant associations were observed for all subgroups.

Some studies found that reduced p16 expression in 55–89% of oral and oropharyngeal SCC [44–46]. The unusual p16 protein expression is probably due to different demographical and clinicopathological characteristics, HPV-driven HNSCC cases, or gene hypermethylation, etc [47–49]. Demokan’s study noted that promoter hypermethylation levels were associated with decreased p16 expression in the tumor samples [22]. Interestingly, Annette’s study found that 95% (110/116) of OSCC did not overexpress p16, while *p16* ^*INK4a*^ promoter methylation was 18% (20/113) [50]. Human papillomaviruses (HPV) is also an important factor in carcinogenesis of HNSCC. It is well documented that HPV infection induced overexpression p16 in OSCCs [51–54], however, Weiss’s study did not show any significantly difference frequencies of *p16* ^*INK4a*^ promoter methylation between HPV16 positive cancer and HPV16 negative cancer [24]. Then, future studies are needed to evaluate the interaction of HPV16 infection and p16INK4a promoter methylation on expression p16 in HNSCC.

There were some limitations in the meta-analysis. Firstly, a significant heterogeneity across the studies was found (*I* ^*2*^ = 60.1%, Q = 50.07, *P*<0.001) in the meta-analysis. According to meta-regression and subgroup analysis, no sources of heterogeneity were founded. Therefore, some source of heterogeneity may exist among studies. Secondly, we do not study the association between *p16* ^*INK4a*^ promoter methylation and disease characteristics (stage, metastasis, relapse and so on) of HNSCC. According to study the association between *p16* ^*INK4a*^ promoter methylation and disease characteristics, the special role of *p16* ^*INK4a*^ promoter methylation in the carcinogenic process of HNSCC may be found.

In conclusion, the results of the meta-analysis showed aberrant methylation of *p16* ^*INK4a*^ promoter was found to be associated with HNSCC, which suggested that promoter methylation of *p16* ^*INK4a*^ may be a potential biomarker in the carcinogenic process of HNSCC.

## Supporting Information

S1 Checklist PRISMA Checklist. (DOCX)

S2 Checklist Meta-analysis on Genetic Association Studies Checklist. (DOCX)

## References

[1] Jemal A, Bray F, Center MM, Ferlay J, Ward E, Forman D. Global cancer statistics. CA: a cancer journal for clinicians. 2011;61(2):69–90. Epub 2011/02/08. 10.3322/caac.20107 . 21296855

[2] Leemans CR, Braakhuis BJ, Brakenhoff RH. The molecular biology of head and neck cancer. Nature reviews Cancer. 2011;11(1):9–22. Epub 2010/12/17. 10.1038/nrc2982 . 21160525

[3] Scully C, Field JK, Tanzawa H. Genetic aberrations in oral or head and neck squamous cell carcinoma (SCCHN): 1. Carcinogen metabolism, DNA repair and cell cycle control. Oral Oncol. 2000;36(3):256–63. Epub 2000/05/04. . 10793327 10.1016/s1368-8375(00)00007-5

[4] Lukas J, Parry D, Aagaard L, Mann DJ, Bartkova J, Strauss M, et al Retinoblastoma-protein-dependent cell-cycle inhibition by the tumour suppressor p16. Nature. 1995;375(6531):503–6. Epub 1995/06/08. 10.1038/375503a0 . 7777060

[5] Piepkorn M. Melanoma genetics: an update with focus on the CDKN2A(p16)/ARF tumor suppressors. Journal of the American Academy of Dermatology. 2000;42(5 Pt 1):705–22; quiz 23–6. Epub 2000/04/25. . 10775844 10.1067/mjd.2000.104687

[6] Nobori T, Miura K, Wu DJ, Lois A, Takabayashi K, Carson DA. Deletions of the cyclin-dependent kinase-4 inhibitor gene in multiple human cancers. Nature. 1994;368(6473):753–6. Epub 1994/04/21. 10.1038/368753a0 . 8152487

[7] Okamoto A, Demetrick DJ, Spillare EA, Hagiwara K, Hussain SP, Bennett WP, et al Mutations and altered expression of p16INK4 in human cancer. Proc Natl Acad Sci U S A. 1994;91(23):11045–9. Epub 1994/11/08. 7972006 10.1073/pnas.91.23.11045 PMC45163

[8] de Assis LV, Locatelli J, Isoldi MC. The role of key genes and pathways involved in the tumorigenesis of Malignant Mesothelioma. Biochimica et biophysica acta. 2014;1845(2):232–47. Epub 2014/02/05. 10.1016/j.bbcan.2014.01.008 . 24491449

[9] Higgins JP, Thompson SG. Quantifying heterogeneity in a meta-analysis. Statistics in medicine. 2002;21(11):1539–58. Epub 2002/07/12. 10.1002/sim.1186 . 12111919

[10] Begg CB, Mazumdar M. Operating characteristics of a rank correlation test for publication bias. Biometrics. 1994;50(4):1088–101. Epub 1994/12/01. . 7786990

[11] Egger M, Davey Smith G, Schneider M, Minder C. Bias in meta-analysis detected by a simple, graphical test. BMJ (Clinical research ed). 1997;315(7109):629–34. Epub 1997/10/06. 9310563 10.1136/bmj.315.7109.629 PMC2127453

[12] R R. The file drawer problem and tolerance for null results. Psychological bulletin. 1979;(86):638–41.

[13] Laytragoon-Lewin N, Chen F, Castro J, Elmberger G, Rutqvist LE, Lewin F, et al DNA content and methylation of p16, DAPK and RASSF1A gene in tumour and distant, normal mucosal tissue of head and neck squamous cell carcinoma patients. Anticancer research. 2010;30(11):4643–8. Epub 2010/12/01. . 21115918

[14] Steinmann K, Sandner A, Schagdarsurengin U, Dammann RH. Frequent promoter hypermethylation of tumor-related genes in head and neck squamous cell carcinoma. Oncology reports. 2009;22(6):1519–26. Epub 2009/11/04. . 19885608 10.3892/or_00000596

[15] Shaw RJ, Liloglou T, Rogers SN, Brown JS, Vaughan ED, Lowe D, et al Promoter methylation of P16, RAR beta, E-cadherin, cyclin A1 and cytoglobin in oral cancer: quantitative evaluation using pyrosequencing. British journal of cancer. 2006;94(4):561–8. 10.1038/sj.bjc.6602972 PubMed PMID: WOS:000235475300017. 16449996 PMC2361183

[16] Maruya S, Issa JP, Weber RS, Rosenthal DI, Haviland JC, Lotan R, et al Differential methylation status of tumor-associated genes in head and neck squamous carcinoma: incidence and potential implications. Clinical cancer research: an official journal of the American Association for Cancer Research. 2004;10(11):3825–30. Epub 2004/06/03. 10.1158/1078-0432.ccr-03-0370 . 15173091

[17] Nakahara Y, Shintani S, Mihara M, Ueyama Y, Matsumura T. High frequency of homozygous deletion and methylation of p16(INK4A) gene in oral squamous cell carcinomas. Cancer Letters. 2001;163(2):221–8. 10.1016/s0304-3835(00)00699-6 PubMed PMID: WOS:000168440600011. 11165758

[18] Sanchez-Cespedes M, Esteller M, Wu L, Nawroz-Danish H, Yoo GH, Koch WM, et al Gene promoter hypermethylation in tumors and serum of head and neck cancer patients. Cancer research. 2000;60(4):892–5. Epub 2000/03/08. . 10706101

[19] Kis A, Tatar TZ, Gall T, Boda R, Tar I, Major T, et al. Frequency of Genetic and Epigenetic Alterations of p14ARF and p16INK4A in Head and Neck Cancer in a Hungarian Population. Pathology oncology research: POR. 2014. Epub 2014/04/09. 10.1007/s12253-014-9775-9 . 24710824

[20] Bhatia V, Goel MM, Makker A, Tewari S, Yadu A, Shilpi P, et al. Promoter Region Hypermethylation and mRNA Expression of MGMT and p16 Genes in Tissue and Blood Samples of Human Premalignant Oral Lesions and Oral Squamous Cell Carcinoma. Biomed Research International. 2014. 10.1155/2014/248419 PubMed PMID: WOS:000337398400001. PMC4058681 24991542

[21] Dang J, Bian Y-Q, Sun JY, Chen F, Dong G-Y, Liu Q, et al MicroRNA-137 promoter methylation in oral lichen planus and oral squamous cell carcinoma. Journal of Oral Pathology & Medicine. 2013;42(4):315–21. 23121285 10.1111/jop.12012

[22] Demokan S, Chuang A, Suoglu Y, Ulusan M, Yalniz Z, Califano JA, et al Promoter methylation and loss of p16(INK4a) gene expression in head and neck cancer. Head & neck. 2012;34(10):1470–5. Epub 2011/11/23. 10.1002/hed.21949 . 22106032

[23] Wong YK, Lee LT, Liu CJ. Hypermethylation of MGMT and DAPK gene promoters is associated with tumorigenesis and metastasis in oral squamous cell carcinoma. Journal of Dental Sciences. 2011;6(3):158–64. 10.1016/j.jds.2011.05.006 PubMed PMID: WOS:000295905700006.

[24] Weiss D, Basel T, Sachse F, Braeuninger A, Rudack C. Promoter Methylation of Cyclin A1 Is Associated With Human Papillomavirus 16 Induced Head and Neck Squamous Cell Carcinoma Independently of p53 Mutation. Molecular Carcinogenesis. 2011;50(9):680–8. 10.1002/mc.20798 PubMed PMID: WOS:000293952200003. 21563216

[25] Kaur J, Demokan S, Tripathi SC, Macha MA, Begum S, Califano JA, et al Promoter hypermethylation in Indian primary oral squamous cell carcinoma. International journal of cancer Journal international du cancer. 2010;127(10):2367–73. Epub 2010/05/18. 10.1002/ijc.25377 20473870 PMC2946507

[26] Su PF, Huang WL, Wu HT, Wu CH, Liu TY, Kao SY. p16(INK4A) promoter hypermethylation is associated with invasiveness and prognosis of oral squamous cell carcinoma in an age-dependent manner. Oral Oncology. 2010;46(10):734–9. 10.1016/j.oraloncology.2010.07.002 PubMed PMID: WOS:000282281600005. 20729138

[27] Ghosh A, Ghosh S, Maiti GP, Sabbir MG, Alam N, Sikdar N, et al SH3GL2 and CDKN2A/2B loci are independently altered in early dysplastic lesions of head and neck: correlation with HPV infection and tobacco habit. The Journal of pathology. 2009;217(3):408–19. Epub 2008/11/22. 10.1002/path.2464 . 19023882

[28] Martone T, Gillio-Tos A, De Marco L, Fiano V, Maule M, Cavalot A, et al Association between hypermethylated tumor and paired surgical margins in head and neck squamous cell carcinomas. Clinical cancer research: an official journal of the American Association for Cancer Research. 2007;13(17):5089–94. Epub 2007/09/06. 10.1158/1078-0432.ccr-07-0119 . 17785562

[29] Kulkarni V, Saranath D. Concurrent hypermethylation of multiple regulatory genes in chewing tobacco associated oral squamous cell carcinomas and adjacent normal tissues. Oral Oncology. 2004;40(2):145–53. 10.1016/s1368-8375(03)00143-x PubMed PMID: WOS:000188399100005. 14693237

[30] Wong TS, Man MW, Lam AK, Wei WI, Kwong YL, Yuen AP. The study of p16 and p15 gene methylation in head and neck squamous cell carcinoma and their quantitative evaluation in plasma by real-time PCR. European journal of cancer (Oxford, England: 1990). 2003;39(13):1881–7. Epub 2003/08/23. . 12932666 10.1016/s0959-8049(03)00428-3

[31] Weber A, Wittekind C, Tannapfel A. Genetic and epigenetic alterations of 9p21 gene products in benign and malignant tumors of the head and neck. Pathology, research and practice. 2003;199(6):391–7. Epub 2003/08/20. 10.1078/0344-0338-00435 . 12924439

[32] Rosas SL, Koch W, da Costa Carvalho MG, Wu L, Califano J, Westra W, et al Promoter hypermethylation patterns of p16, O6-methylguanine-DNA-methyltransferase, and death-associated protein kinase in tumors and saliva of head and neck cancer patients. Cancer research. 2001;61(3):939–42. Epub 2001/02/28. . 11221887

[33] Righini CA, de Fraipont F, Timsit JF, Faure C, Brambilla E, Reyt E, et al Tumor-specific methylation in saliva: a promising biomarker for early detection of head and neck cancer recurrence. Clinical cancer research: an official journal of the American Association for Cancer Research. 2007;13(4):1179–85. Epub 2007/02/24. 10.1158/1078-0432.ccr-06-2027 . 17317827

[34] Song B, Ai J, Kong X, Liu D, Li J. Aberrant DNA Methylation of P16, MGMT, and hMLH1 Genes in Combination with MTHFR C677T Genetic Polymorphism in gastric cancer. Pak J Med Sci. 2013;29(6):1338–43. Epub 2014/02/20. 24550949 10.12669/pjms.296.3711 PMC3905372

[35] Liggett WH Jr., Sidransky D. Role of the p16 tumor suppressor gene in cancer. Journal of clinical oncology: official journal of the American Society of Clinical Oncology. 1998;16(3):1197–206. Epub 1998/03/21. . 9508208 10.1200/JCO.1998.16.3.1197

[36] Belinsky SA, Klinge DM, Dekker JD, Smith MW, Bocklage TJ, Gilliland FD, et al Gene promoter methylation in plasma and sputum increases with lung cancer risk. Clinical cancer research: an official journal of the American Association for Cancer Research. 2005;11(18):6505–11. Epub 2005/09/17. 10.1158/1078-0432.ccr-05-0625 . 16166426

[37] Hsu LS, Lee HC, Chau GY, Yin PH, Chi CW, Lui WY. Aberrant methylation of EDNRB and p16 genes in hepatocellular carcinoma (HCC) in Taiwan. Oncology reports. 2006;15(2):507–11. Epub 2006/01/05. . 16391877

[38] Mino A, Onoda N, Yashiro M, Aya M, Fujiwara I, Kubo N, et al Frequent p16 CpG island hypermethylation in primary remnant gastric cancer suggesting an independent carcinogenic pathway. Oncology reports. 2006;15(3):615–20. Epub 2006/02/09. . 16465421

[39] Askari M, Sobti RC, Nikbakht M, Sharma SC. Promoter hypermethylation of tumour suppressor genes (p14/ARF and p16/INK4a): case-control study in North Indian population. Molecular biology reports. 2013;40(8):4921–8. Epub 2013/05/29. 10.1007/s11033-013-2592-5 . 23712779

[40] Herman JG, Graff JR, Myohanen S, Nelkin BD, Baylin SB. Methylation-specific PCR: a novel PCR assay for methylation status of CpG islands. Proc Natl Acad Sci U S A. 1996;93(18):9821–6. Epub 1996/09/03. 8790415 10.1073/pnas.93.18.9821 PMC38513

[41] Toyota M, Ho C, Ahuja N, Jair KW, Li Q, Ohe-Toyota M, et al Identification of differentially methylated sequences in colorectal cancer by methylated CpG island amplification. Cancer research. 1999;59(10):2307–12. Epub 1999/05/27. . 10344734

[42] Yates DR, Rehman I, Meuth M, Cross SS, Hamdy FC, Catto JW. Methylational urinalysis: a prospective study of bladder cancer patients and age stratified benign controls. Oncogene. 2006;25(13):1984–8. Epub 2005/11/17. 10.1038/sj.onc.1209209 . 16288222

[43] Chen Y, Li J, Yu X, Li S, Zhang X, Mo Z, et al APC gene hypermethylation and prostate cancer: a systematic review and meta-analysis. European journal of human genetics: EJHG. 2013;21(9):929–35. Epub 2013/01/10. 10.1038/ejhg.2012.281 23299921 PMC3746257

[44] Pande P, Mathur M, Shukla NK, Ralhan R. pRb and p16 protein alterations in human oral tumorigenesis. Oral oncology. 1998;34(5):396–403. . 9861348 10.1016/s1368-8375(98)00024-4

[45] El-Naggar AK, Lai S, Clayman GL, Zhou JH, Tucker SA, Myers J, et al Expression of p16, Rb, and cyclin D1 gene products in oral and laryngeal squamous carcinoma: biological and clinical implications. Human pathology. 1999;30(9):1013–8. . 10492034 10.1016/s0046-8177(99)90217-4

[46] Wu CL, Roz L, McKown S, Sloan P, Read AP, Holland S, et al DNA studies underestimate the major role of CDKN2A inactivation in oral and oropharyngeal squamous cell carcinomas. Genes, chromosomes & cancer. 1999;25(1):16–25. . 10221335

[47] Hasegawa M, Nelson HH, Peters E, Ringstrom E, Posner M, Kelsey KT. Patterns of gene promoter methylation in squamous cell cancer of the head and neck. Oncogene. 2002;21(27):4231–6. 10.1038/sj.onc.1205528 . 12082610

[48] O'Regan EM, Toner ME, Finn SP, Fan CY, Ring M, Hagmar B, et al p16(INK4A) genetic and epigenetic profiles differ in relation to age and site in head and neck squamous cell carcinomas. Human pathology. 2008;39(3):452–8. 10.1016/j.humpath.2007.08.004 . 18261630

[49] Ai L, Stephenson KK, Ling W, Zuo C, Mukunyadzi P, Suen JY, et al The p16 (CDKN2a/INK4a) tumor-suppressor gene in head and neck squamous cell carcinoma: a promoter methylation and protein expression study in 100 cases. Modern pathology: an official journal of the United States and Canadian Academy of Pathology, Inc. 2003;16(9):944–50. 10.1097/01.MP.0000085760.74313.DD . 13679459

[50] Lim AM, Do H, Young RJ, Wong SQ, Angel C, Collins M, et al Differential mechanisms of CDKN2A (p16) alteration in oral tongue squamous cell carcinomas and correlation with patient outcome. International journal of cancer Journal international du cancer. 2014;135(4):887–95. 10.1002/ijc.28727 . 24436120

[51] Fakhry C, Westra WH, Li S, Cmelak A, Ridge JA, Pinto H, et al Improved survival of patients with human papillomavirus-positive head and neck squamous cell carcinoma in a prospective clinical trial. J Natl Cancer Inst. 2008;100(4):261–9. Epub 2008/02/14. 10.1093/jnci/djn011 . 18270337

[52] Smeets SJ, Hesselink AT, Speel EJ, Haesevoets A, Snijders PJ, Pawlita M, et al A novel algorithm for reliable detection of human papillomavirus in paraffin embedded head and neck cancer specimen. International journal of cancer Journal international du cancer. 2007;121(11):2465–72. Epub 2007/08/08. 10.1002/ijc.22980 . 17680565

[53] Klussmann JP, Gultekin E, Weissenborn SJ, Wieland U, Dries V, Dienes HP, et al Expression of p16 protein identifies a distinct entity of tonsillar carcinomas associated with human papillomavirus. The American journal of pathology. 2003;162(3):747–53. Epub 2003/02/25. 10.1016/s0002-9440(10)63871-0 12598309 PMC1868106

[54] Begum S, Cao D, Gillison M, Zahurak M, Westra WH. Tissue distribution of human papillomavirus 16 DNA integration in patients with tonsillar carcinoma. Clinical cancer research: an official journal of the American Association for Cancer Research. 2005;11(16):5694–9. Epub 2005/08/24. 10.1158/1078-0432.ccr-05-0587 . 16115905

